# T Cell Responses to Neural Autoantigens Are Similar in Alzheimer’s Disease Patients and Age-Matched Healthy Controls

**DOI:** 10.3389/fnins.2020.00874

**Published:** 2020-08-27

**Authors:** Rekha Dhanwani, John Pham, Ashmitaa Logandha Ramamoorthy Premlal, April Frazier, Atul Kumar, Maria Elena Pero, Francesca Bartolini, Juliana Rezende Dutra, Karen S. Marder, Bjoern Peters, David Sulzer, Alessandro Sette, Cecilia S. Lindestam Arlehamn

**Affiliations:** ^1^Division of Vaccine Discovery, La Jolla Institute for Immunology, La Jolla, CA, United States; ^2^Department of Pathology and Cell Biology, Columbia University, New York, NY, United States; ^3^Department of Veterinary Medicine and Animal Production, University of Naples Federico II, Naples, Italy; ^4^Department of Neurology, Columbia University Irving Medical Center, New York, NY, United States; ^5^Department of Medicine, University of California, San Diego, La Jolla, CA, United States; ^6^Department of Neurology, New York State Psychiatric Institute, Columbia University, New York, NY, United States; ^7^Department of Psychiatry and Pharmacology, New York State Psychiatric Institute, Columbia University, New York, NY, United States

**Keywords:** Alzheimer’s disease, neurodegenration, autoimmunity, T cell responses, transcriptomics, neuroantigens

## Abstract

Alzheimer’s disease (AD), a chronic multifactorial and complex neurodegenerative disorder is a leading cause of dementia. Recently, neuroinflammation has been hypothesized as a contributing factor to AD pathogenesis. The role of adaptive immune responses against neuronal antigens, which can either confer protection or induce damage in AD, has not been fully characterized. Here, we measured T cell responses to several potential antigens of neural origin including amyloid precursor protein (APP), amyloid beta (Aβ), tau, α-synuclein, and transactive response DNA binding protein (TDP-43) in patients with AD and age-matched healthy controls (HC). Antigen-specific T cell reactivity was detected for all tested antigens, and response to tau-derived epitopes was particularly strong, but no significant differences between individuals with AD and age-matched HC were identified. We also did not observe any correlation between the antigen-specific T cell responses and clinical variables including age, gender, years since diagnosis and cognitive score. Additionally, further characterization did not reveal any differences in the relative frequency of major Peripheral Blood Mononuclear Cells (PBMC) subsets, or in the expression of genes between AD patients and HC. These observations have not identified a key role of neuronal antigen-specific T cell responses in AD.

## Introduction

Alzheimer’s disease (AD) is a neurodegenerative disorder associated with the progressive loss of structure and function in neurons, leading to dementia and affecting predominantly elderly individuals. The disease is characterized by extracellular plaques that consist of amyloid beta peptides (Aβ) that is produced from the amyloid precursor protein (APP) and neurofibrillary tangles that consist of hyperphosphorylated tau (Grundke-Iqbal et al., 1986; Butterfield and Boyd-Kimball, 2004).

Aggregation or misfolding of autoantigens expressed in the brain, such as Aβ (Finder and Glockshuber, 2007), α-synuclein (Paleologou et al., 2005), tau (Honson and Kuret, 2008), and transactive response DNA binding protein (TDP-43) (Cook et al., 2008; Guo et al., 2011; Herman et al., 2011; Jiang et al., 2016), could render susceptibility to adaptive T cell responses and are associated with Parkinson’s disease (PD), AD, and amyotrophic lateral sclerosis (ALS). The role of T cell autoimmunity has been studied in various animal models (Wong et al., 2002; Bloom et al., 2005; Janus and Welzl, 2010; Dawson et al., 2018), but less frequently in humans (Sulzer et al., 2017; Lodygin et al., 2019).

There are, however, increasing reasons to speculate that T cell responses in neurodegenerative diseases, including AD, are mounted against aggregated or misfolded neural proteins (Mietelska-Porowska and Wojda, 2017). Some studies have shown increased infiltration of T cells in response to inflammatory signals in the brains of AD patients (Itagaki et al., 1988; Pirttila et al., 1992; Merlini et al., 2018). It is also speculated that neuroinflammation is not the only source of neurodegeneration in the AD brain. The AD induced neurodegeneration could emerge due to multi−faceted interactions between inflammation and other processes such as NFT formation, Aβ deposition, glutamate excitotoxicity, reactive oxygen intermediate toxicity, and/or other mechanisms that induce neuronal death in the AD cortex (Cooper et al., 2000). However, the functional role and reactivity of the CNS infiltrating T cells have not been determined, in part due to limited sample availability. An alternative approach is to study T cell responses in PBMCs that can help better understand the role of adaptive immune responses in AD in more accessible samples. This approach was recently successful in characterizing the role of T cells in AD (Monsonego et al., 2003; Gate et al., 2020), as well as Parkinson’s disease (PD), another important neurodegenerative disorder (Sulzer et al., 2017; Lindestam Arlehamn et al., 2019, 2020).

In addition to self-antigens implicated in the pathogenesis of neurodegenerative diseases, some microbes like *Bordetella pertussis* (PT) and herpesviruses have also been hypothesized to be associated with the development of AD (Lin et al., 2002; Rubin and Glazer, 2017; Allnutt et al., 2020). Therefore, characterizing neural and microbial antigen-specific T cell responses in peripheral T cells from individuals with AD may help untangle the complex concept of autoimmunity in neurodegeneration and establish a correlation between T cell reactivity and disease progression.

Here, to assess the potential involvement of peripheral T cells in AD, we performed a range of immunological assays in individuals with AD and age-matched HC. Specifically, we (i) compared the relative frequency of major PBMC cell subsets, (ii) characterized T cell responses to proteins involved in neurodegeneration such as Aβ, APP, tau, α-synuclein, TDP-43, PT, and Epstein-Barr virus and cytomegalovirus (EBV/CMV), (iii) correlated antigen-specific reactivity with demographic and clinical variables including age, gender, time since diagnosis and cognitive score, and (iv) conducted a transcriptomic analysis of PBMC, CD4 memory and CD8 memory T cells to assess differential expression of genes in AD compared to HC. In summary, these analyses revealed no statistically significant differences between the populations of AD patients and age-matched HC.

## Results

### Relative Frequency of Major PBMC Subsets in AD Compared to Age-Matched HC

**FIGURE 1:**
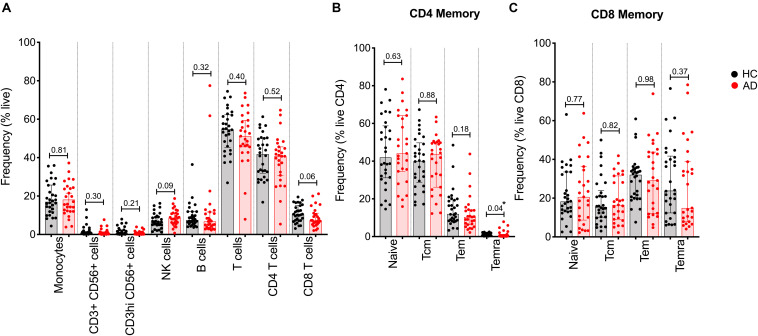
Relative frequency of different cell subsets in HC and AD. **(A)** Frequency of major PBMC subsets in AD (red bars and circles) and age-matched HC (black bar and circles). **(B)** CD4 memory and **(C)** CD8 memory T cells were further evaluated for frequency of naïve, effector memory (T_em_), central memory (T_cm_), and T_EMRA_ populations. Each point represents a donor. Median ± interquartile range is displayed. Two-tailed Mann–Whitney test. Cells were gated according to the gating strategy in Supplementary Figure S1.

We previously described the establishment of a flow cytometry panel designed to quantitate the relative frequency of major PBMC subsets in order to examine potential differences as a function of disease states (Burel et al., 2017). Here, we utilized this panel to specifically examine whether differences in lymphocyte subsets could be associated with AD. We first analyzed the relative frequency of major PBMC subsets, i.e., monocytes, NK cells, B cells, T cells, and CD4 and CD8 memory T cells, in 27 AD and 30 age-matched HC by flow cytometric analysis (gating strategy in Supplementary Figure S1). In general, the frequency of all PBMC subsets was remarkably similar between AD and HC (Figure 1). The only significant difference observed was related to the frequency of the T_EMRA_ subset of CD4 memory T cells, which was found to be decreased in AD patients.

### Cytokine Responses to Neural and Microbial Antigens in AD and Age-Matched HC

Aβ, α-synuclein, tau and TDP-43 have been implicated in AD and other forms of dementia, as well as in PD (Paleologou et al., 2005; Finder and Glockshuber, 2007; Cook et al., 2008; Honson and Kuret, 2008; Guo et al., 2011; Herman et al., 2011; Jiang et al., 2016). We examined whether T cell reactivity against these proteins could be detected and, if so, whether differences existed between AD patients and age-matched HC. Accordingly, PBMCs were stimulated for 2 weeks *in vitro* with peptide pools representing the different proteins. The APP pool corresponded to 153 peptides, while the amyloid beta-42 (Aβ) pool encompassed 9 peptides. The previously described α-syn epitope and tau peptide pools consisted of 11 and 70 peptides, respectively (Sulzer et al., 2017; Lindestam Arlehamn et al., 2019). We also studied a TDP-43 peptide pool which is composed of 82 peptides. We further measured cytokine responses against pertussis (PT) and EBV/CMV peptide pools (Bancroft et al., 2016; Dan et al., 2016; Tian et al., 2017; da Silva Antunes et al., 2018).

**FIGURE 2:**
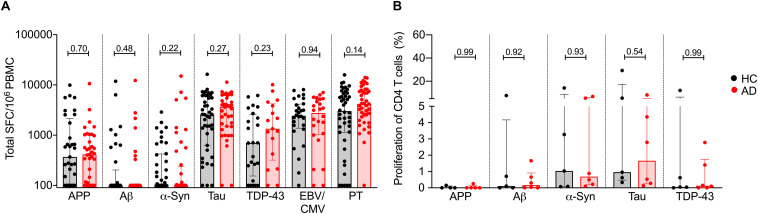
T cell reactivity to APP, Aβ, α-synuclein, tau, TDP-43, EBV/CMV, and PT in AD and age-matched HC. **(A)** Magnitude of total response (sum of IFNγ, IL-5, and IL-10) in HC (black bar and circles) and AD (red bar and circles) to APP (HC, *n* = 33; AD *n* = 37), Aβ (HC, *n* = 37; AD, *n* = 37), α-syn (HC, *n* = 40; AD, *n* = 44), tau (HC, *n* = 43; AD *n* = 41), TDP-43 (HC, *n* = 24; AD, *n* = 17), EBV/CMV (HC, *n* = 28; AD, *n* = 24), and PT (HC, *n* = 44; AD, *n* = 43). Each dot represents a subject. Median ± interquartile range is shown. Two-tailed Mann–Whitney test. **(B)** Proliferation of CD4 T cells in AD (*n* = 6) and HC (*n* = 6). % of proliferated CD4^++^ T cell (CFSE-ve) population in DMSO stimulated condition was subtracted from antigen stimulated condition. Median ± interquartile range is displayed. Each point represents a subject. Two-tailed Mann–Whitney test.

**FIGURE 3:**
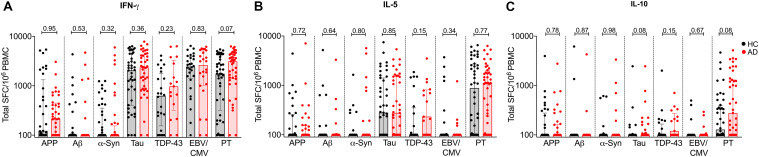
Individual cytokine responses to APP, Aβ, α-synuclein, tau, TDP-43, EBV/CMV, and PT in AD and age-matched HC **(A)** IFNγ, **(B)** IL-5, and **(C)** IL-10. Each symbol represents a subject. HC is represented in black bar and circles and AD is represented in red bar and circles. Median ± interquartile range is shown.

After 2 weeks, cultures were harvested and stimulated with the relevant antigen used in establishing the cultures, or PHA as a control, in a triple-color IFNγ, IL-5, and IL-10 Fluorospot assay. While T cell reactivity was detected for all tested antigens, no significant differences in total response magnitude (Figure 2A) or specific cytokines (Figure 3) were observed between AD patients and age-matched HC.

Additionally, due to the difference in ethnicity between AD and HC cohorts (Table 1), we also analyzed our data excluding non-Caucasian subjects and found no significant difference in T cell reactivity between AD and HC, with the exception of PT reactivity. PT-specific T cell responses were higher in AD as compared to HC (Supplementary Figure S2). A trend for higher PT-specific T cell reactivity was also observed in the entire cohort (Figure 2).

**TABLE 1:** Characteristics of the subjects enrolled in the study.

Characteristics and demographics	AD	HC
Total subjects enrolled	51	53
Median age (range), yr	69, (52–89)	68, (56–92)
Male, % (n)	51% (26)	43% (23)
Caucasian, % (n)	65% (33)	92.5% (49)
Median years since diagnosis, (range), yr	4, (0.5–11)	NA
Median MoCA^a^ (range)	18 (8–26)	28 (24–30)
Median MMSE^b^ (range)	22 (16–28)	30 (29–30)

*^*a*^MoCA collected at CUMC only. ^*b*^MMSE collected at PrecisionMed only.*

We have previously described higher magnitude of responses against tau as compared to α-syn in individuals with PD and HC (Lindestam Arlehamn et al., 2019). Notably, the magnitude of cytokine responses against APP, Aβ and α-syn were weaker than that observed against tau, EBV/CMV and PT. This suggests that these antigens are less immunogenic.

### Proliferative Responses in AD and Age-Matched HC

We next sought to confirm these findings using an alternative readout, namely a proliferative assay that was recently described as a means to detect β-synuclein T cell reactivity in multiple sclerosis and PD patients (Lodygin et al., 2019). Accordingly, we determined the frequency of proliferating CD4^+^ T cells in response to the same antigens, in a subset of 6 AD and 6 HC. The PBMCs were stained with CFSE, stimulated with the respective antigens and cultured for 11 days. After 11 days, cells were stained with CD3, CD4, and CD8 antibodies, and the percentage of CD4^+^ T cell proliferation was measured (gating strategy in Supplementary Figure S3). Utilizing this alternative readout, no difference was observed in the percentage of proliferating CD4^+^ T cells between AD and HC (Figure 2B).

### Correlations Between Antigen Specific T Cell Reactivity, Sex, and Clinical Variables

**FIGURE 4:**
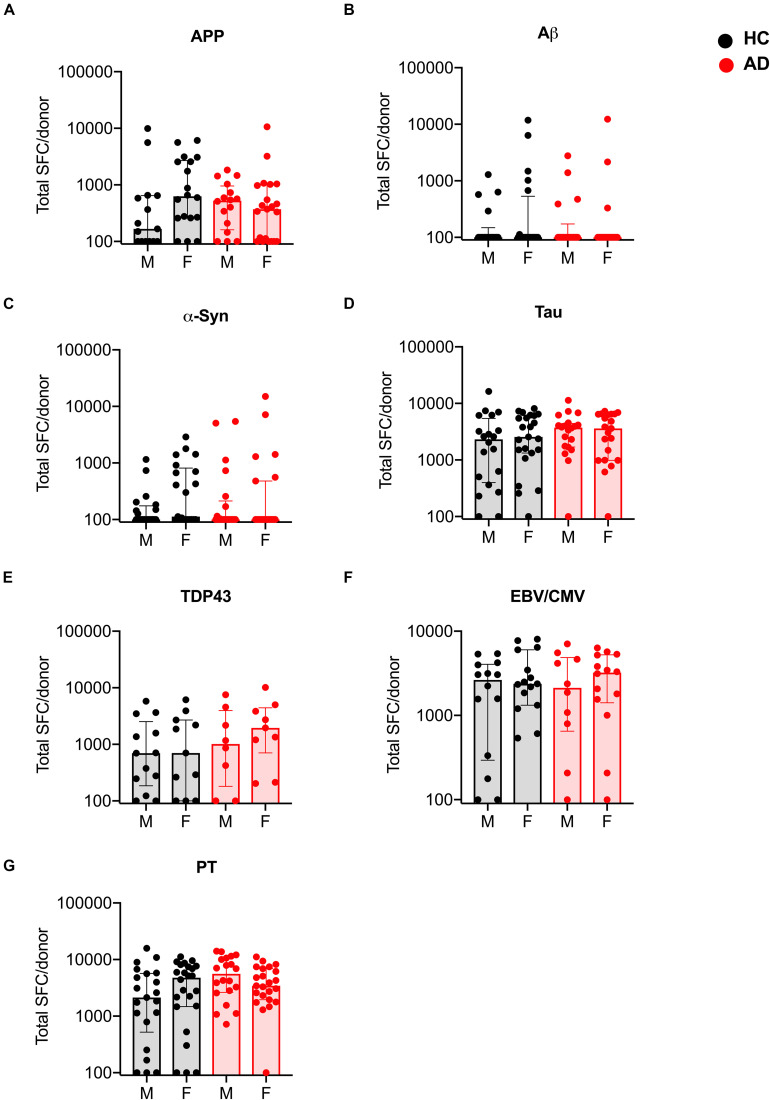
Magnitude of T cell responses to specific antigens in males and females with AD and age-matched HC. **(A)** APP (HC males *n* = 15, females *n* = 18; AD males *n* = 16, females *n* = 21) **(B)** Aβ (HC males *n* = 18, females *n* = 20; AD males *n* = 18, females *n* = 19) **(C)** α-synuclein (HC males *n* = 20, females *n* = 21; AD males *n* = 21, females *n* = 23) **(D)** tau (HC males *n* = 20, females *n* = 23; AD males *n* = 20, females *n* = 21) **(E)** TDP-43 (HC males *n* = 13, females *n* = 11; AD males *n* = 8, females *n* = 9) **(F)** EBV/CMV (HC males *n* = 14, females *n* = 15; AD males *n* = 10, females *n* = 14) **(G)** PT (HC males *n* = 21, females *n* = 24; AD males *n* = 20, females *n* = 23). Each dot represents a donor. Black dot (within gray bar) represents HC and red dot (within red bar) represents AD. Median with interquartile range is displayed.

In a recent study (Lindestam Arlehamn et al., 2020), the T cell reactivity to α-synuclein was related to early diagnoses of PD, suggesting that correlational studies may help establish a link with disease progression. To investigate whether any such relationships exists in AD, we examined the possible correlations between antigen specific T cell reactivity and clinical variables such as gender, age, time since diagnosis and cognitive score. First, we compared the magnitude and frequency of antigen-specific T cell responses as a function of sex and found no significant difference in the T cell responses of males and females with AD as compared to age-matched HC (Figure 4).

**FIGURE 5:**
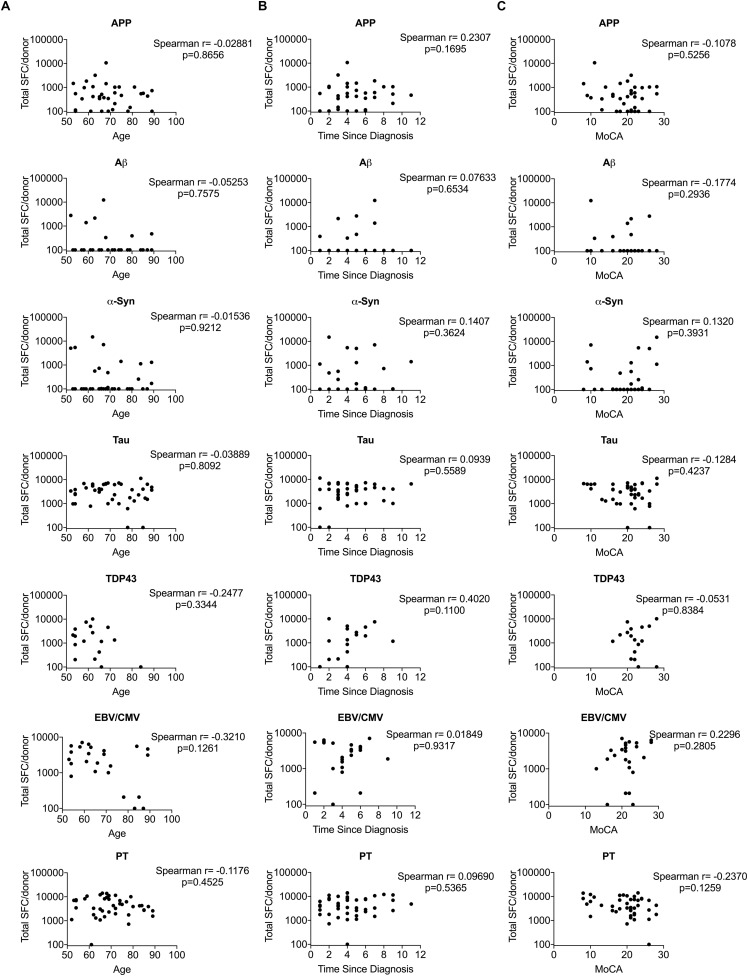
Correlation between T cell reactivity to different antigens and clinical variables. **(A)** Age vs. T cell reactivity. **(B)** Time since diagnosis vs. T cell reactivity **(C)** MoCA vs. T cell reactivity. *X*-axis in panels **(A–C)** represent clinical variables age, time since diagnosis and MoCA, respectively and *Y*-axis represents T cell reactivity. Each dot represents a donor. Non-parametric Spearman test was performed.

Next, we examined whether antigen-specific T cell reactivity correlated with clinical variables relevant to AD patients: age, years since diagnosis and cognitive function (the Montreal Cognitive Assessment; MoCA) (Davis et al., 2015). Unlike Parkinson’s disease, where a strong positive correlation was established between T cell reactivity to α-syn and clinical variables including age and time since diagnosis (Lindestam Arlehamn et al., 2020), no correlation was detected in AD patients between antigen-specific T cell reactivity and clinical parameters (Figure 5).

### Transcriptional Profiling of PBMCs, CD4, and CD8 Memory T Cells in AD and HC

**FIGURE 6:**
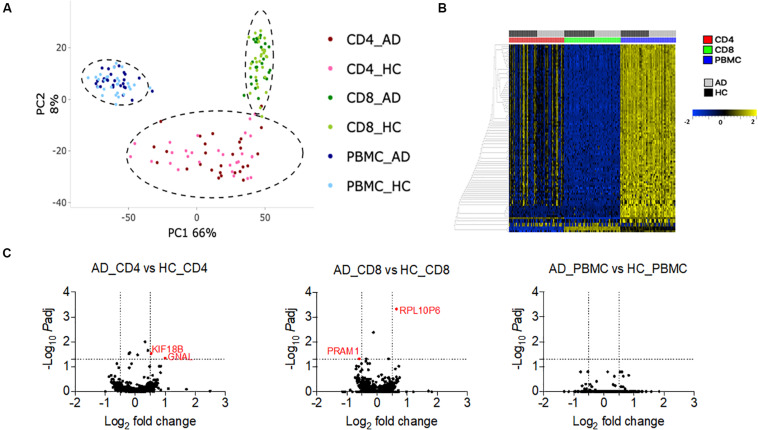
Transcriptomic profile of PBMC, CD4 memory and CD8 memory T cells in AD and HC **(A)**. PCA analysis of gene expression data from PBMC (HC *n* = 28 and AD *n* = 27), CD4 memory (HC *n* = 28 and AD *n* = 27), and CD8 memory (HC *n* = 30 and AD *n* = 26) T cells of HC and AD **(B)**. Heat map of the top 100 variable genes in PBMC, CD4 memory, and CD8 memory T cells **(C)**. Volcano plots show log_2_ fold change vs. –log_10_ p*_*adj*_* value for the comparison between AD and HC PBMC, AD, and HC CD4 memory and AD and HC CD8 memory T cells. Dotted lines indicate the cut off limit of log_2_ fold change (on *x*-axis) and –log_10_ p*_*adj*_* (on *y*-axis). Because most of the genes are non-significant, they fall on the axis. The genes with log_2_ fold change greater than 0.5 or less than –0.5 and adjusted *p*-Value less than 0.05 are considered significant and are represented in red.

Finally, we examined whether differences between individuals with AD and HC could be detected at the level of gene expression in different cell populations. PBMCs, CD4 memory, and CD8 memory T cells from the CUMC cohort were sorted (gating strategy in Supplementary Figure S1) and RNA was extracted as described in the methods section. Principal component analysis (PCA) of the 1000 most variable genes revealed that PBMCs, CD4 memory and CD8 memory T cells formed distinct clusters, as expected (Figure 6A). This cell subset clustering was also evident when the 100 most variable genes were considered (Figure 6B and Supplementary Table S1). Next, we performed a pairwise analysis to identify any differentially expressed (DE) genes between AD and HC in PBMCs, CD4 memory and CD8 memory T cells. At a cut-off of log_2_ fold change >0.5 and p*_*adj*_* less than 0.05, PBMCs had no differences in patterns of gene expression between AD and HC (0 DE genes), and only two genes each were differentially expressed in CD4 memory (GNAL and KIF18B) and CD8 memory (RPL10P6 and PRAM1) T cells (Figure 6C). Moreover, we exclusively looked into differential expression of miRNAs due to their wide implication in AD pathogenesis (Cosin-Tomas et al., 2017; Hara et al., 2017; Kumar and Reddy, 2018; Moradifard et al., 2018; Angelucci et al., 2019). However, as shown in Supplementary Figure S4, there were no miRNAs that were differentially expressed between AD and HC subjects in either of the cell subsets tested in this study, reflecting the lack of differences observed in the overall DEseq analysis.

## Discussion

We investigated autoreactivity of T cells against antigens that have been associated with AD, specifically Aβ, APP, α-synuclein, tau and TDP-43. To do so, peripheral T cells of AD patients and age-matched HC were systematically compared for differences in the frequency of T cell subsets, cytokine responses, proliferative capacity and differential gene expression signatures.

We chose these targets because they are proteins that accumulate in association with several neurodegenerative diseases, including AD, and are subject to post-translational modifications in the course of disorders that may produce neoantigens. Pathological manifestations are characterized by the immune system’s failure to clear the deposited aggregated proteins (Irvine et al., 2008). This pathological presentation is attributed to a combination of molecular and environmental factors, such as aging and genetics (Cacabelos et al., 2005). At the molecular level, the neurodegenerative diseases are characterized by the accumulation of protein fragments that cluster together, producing toxic effects on neurons and disrupting cell to cell communication.

Several research groups have reported the presence of autoantibodies against a variety of molecules in AD (Du et al., 2001; Rosenmann et al., 2006; Dodel et al., 2011). Natural autoantibodies are produced under physiological conditions to clear dead cells or toxic autoantigens and thereby dampen inflammatory signals. This feature of natural autoantibodies highlights their potential role in conferring protection against the progression of AD (Du et al., 2001; Weksler et al., 2002; Brettschneider et al., 2005; Britschgi et al., 2009; Dodel et al., 2011; Qu et al., 2014). Moreover, some autoantibodies have also emerged as potential biomarkers for AD. However, with the exception of some reports (Monsonego et al., 2003; Gate et al., 2020), the role of aberrant T cell responses that equally contribute to driving autoimmunity in AD has not been well characterized. Thus, addressing the potential role of autoreactive T cells in AD might improve our understanding of neurodegenerative diseases and offer novel avenues of therapeutic intervention.

Here, multiple approaches of investigating T cell reactivity to the various antigens revealed no difference between AD and age-matched healthy controls except near significant increase in IFNγ and IL-10 response to PT in AD patients. This is in agreement with the recent hypothesis that B. pertussis plays a role in the etiology of AD (Rubin and Glazer, 2017). Our findings are consistent with reports of increased T cell reactivity to Aβ in older humans and patients with AD compared to healthy young adults, but no difference in T cell reactivity between healthy older humans and AD (Giubilei et al., 2003; Monsonego et al., 2003). Additionally, no difference was observed in the frequency of various cell subsets, except for CD4 T_EMRA_ subset. A previous study (Richartz-Salzburger et al., 2007) reported only slight increase in circulating CD4^+^ T cells and a decreased frequency of CD3^+^ T cells, CD8^+^ T cells, and CD19^+^ cells in AD compared to healthy controls. Furthermore, recently an increase in CD3^+^CD8^+^CD45RA^+^CD27^–^ TEMRA T cells was reported in mild cognitive impairment (MCI) or AD patients (Gate et al., 2020). In our cohort there was no significant difference in the frequency of this cell subset. Other studies have also reported differences in various immune cell subsets such as decreased T cell numbers and changes in the CD4 T cell compartment (Pellicano et al., 2012; Busse et al., 2017). The discrepancies of the different studies could be related to differences in several factors such as T cell stimulation methodology, sample size, drugs used by patients for disease management and/or in the criteria used to define AD subjects. Our flow cytometry panels did not include in-depth analysis of Tregs, and different subsets of Tregs were recently found to be decreased in AD patients compared to HC (Ciccocioppo et al., 2019). Different miRNAs have previously been identified in serum, plasma, CSF, or brain as differentially regulated or expressed between AD and age-matched HC (Cosin-Tomas et al., 2017; Hara et al., 2017; Kumar and Reddy, 2018; Moradifard et al., 2018; Angelucci et al., 2019). No studies have so far shown differentially expressed miRNAs in these cohorts in peripheral T cell subsets or PBMCs. Future studies can confirm whether differences in other cell subsets, such as specifically Treg subsets, immune parameters, or miRNA expression in these cell subsets exist between AD and HC.

Because it is well-known that aging alters the components of innate and adaptive immunity we included age-matched healthy controls in this study. Age-related irreversible alterations correlate with a general increase in propensity to autoimmunity (Maletto et al., 1994; Simon et al., 2015), which may affect antigen-specific T cell responses. The increase in autoimmunity with age was recently supported by our study in Parkinson’s disease where α-syn-specific T cell responses increase as a function of age (Lindestam Arlehamn et al., 2020). One caveat of our study is that the AD and HC cohorts were not matched for race. However, no differences were detected between race-matched AD and HC cohorts. Moreover, if race influences the immunological responses measured we would have expected to see more differences rather than fewer.

Our findings suggest a difference in the disease pathology of PD and AD. PD is associated with an increase in T cell reactivity to α-synuclein (Sulzer et al., 2017; Lindestam Arlehamn et al., 2020). These increased T cell responses to α-synuclein are negatively correlated with time since PD diagnosis (Lindestam Arlehamn et al., 2020). For AD however, with the exception of a minor decrease in the CD4 T_EMRA_ cell population in AD, no difference in T cell reactivity between AD and HC was noted, and no correlation could be established between T cell reactivity and clinical variables, which is in agreement with a previous study (Giubilei et al., 2003). This may reflect a fundamental difference between PD and AD, with T cells and associated inflammation playing a key role in PD, but not AD. In that respect, it is noteworthy that PD incidence is increased in association with other inflammatory autoimmune diseases such as IBD, and that anti-TNF treatment is associated with a reduction in PD incidence (Peter et al., 2018), while no such effect has been reported in the case of AD.

In conclusion, this study highlights a clear difference between the role of T cell mediated immunity in PD and AD. However, the lack of evidence for differential T cell recognition of the antigens we studied in AD does not rule out that other proteins or the same proteins with different post-translational modifications may show differential response. There may further be different forms or stages of AD where such differences could occur. Hence, further studies deconvoluting the autoantigens at the epitope level, including of CNS infiltrating cells, and investigating the role of post-translationally autoantigen modifications such as acetylation and phosphorylation might reveal differences in T cell reactivity between AD and healthy controls providing detailed insights on AD associated autoimmune responses.

## Materials and Methods

### Ethics Statement

All participants provided written informed consent for participation in the study. Ethical approval was obtained from the Institutional review boards at the La Jolla Institute for Immunology (LJI; protocol number VD-155) and the Columbia University Medical Center (CUMC; protocol number IRB-AAAQ9714).

### Study Subjects

We recruited a total of 104 individuals diagnosed with AD (*n* = 51) and age-matched healthy subjects (*n* = 53) in this study. The subjects were recruited from two sites: 66 subjects from Alzheimer’s Disease Research Center at Columbia University Medical Center (CUMC) (AD *n* = 33 and HC *n* = 33) and 38 subjects from a San Diego-based Contract Research Organization, PrecisionMed (AD *n* = 18 and HC *n* = 20). The characteristics of the enrolled subjects are detailed in Table 1.

Subjects recruited at CUMC were diagnosed by neurologists according to the National Institute of Aging and Alzheimer’s Association criteria (McKhann et al., 2011). Fifteen AD subjects had neuropsychological testing only and 18 AD subjects had neuropsychological testing and combinations of positive biomarkers including SPECT scan (*n* = 6), FDG PET scan (*n* = 4), CSF (*n* = 3), or amyloid scan (*n* = 7). They were all followed for at least 2 consecutive visits. Eleven AD subjects started having cognitive symptoms before age 65, and were thus early onset AD. 33 HC subjects were evaluated for at least 2 consecutive years with a normal neurophysiological testing. The neurophysiological testing comprised of MMSE, MoCA, digit forward and backward, logical memory, selective reminding test, fluency (phonemic and semantic), multilingual naming test, global deficit score and neuropsychiatric inventory questionnaire. Some HC were also screened for the same biomarkers as the AD patients. They were all negative, CSF scan was performed in 7 individuals, and amyloid scan in 10.

At PrecisionMed, AD subjects were diagnosed according to NINCDS-ADRDA criteria (McKhann et al., 1984) by a neurologist or internist. The subjects underwent MRI/CT to rule out other causes of cognitive decline and those that were diagnosed with AD exhibited MMSE score ≤ 26, deficit in two or more areas of cognition, progressive worsening of memory and other cognitive functions along with any of other supportive parameters like progressive deterioration of specific cognitive function such as language (aphasia), motor skills (apraxia) and perceptions (agnosia), impaired activities of daily living and altered patterns of behavior, associated symptoms of depression, insomnia, incontinence, delusions, illusions, hallucinations, catastrophic verbal, emotional, or physical outbursts, sexual disorders, and weight loss, plateaus in the course of progression of the illness and /or seizures at advance stage. Of the 18 AD subjects, 14 were homozygous for APOE ε3, three subjects expressed APOE ε2/ε3, and one expressed APOE ε3/ε4. The HC were self-reported and had MMSE of ≥29.

Neither of the cohorts included neuropathological confirmation of AD, hence it is possible that some individuals may present mixed pathology of AD and other forms of dementia, such as limbic-predominant age-related TDP-43 encephalopathy (LATE), as LATE can mimic AD presentation. However, clinical and imaging or biological tools were used to try to minimize this risk. Moreover, LATE primarily affects older individuals (>80yo). In the CUMC cohort, 10 out of 33 AD subjects were older than 80 years, and all of them had clinical presentation and neuropsychological testing suggestive of AD. In the PrecisionMed cohort, one AD subject was above 80 years.

Another neurological condition that is characterized by deposits of Aβ peptide in the vessels is cerebral amyloid angiopathy (CAA). The deposits in CAA are biochemically similar to the material comprising senile plaques in AD, however subjects with a MRI suggestive of CAA (multiple hemorrhages; single lobar, cortical or cortical-subcortical hemorrhage; superficial siderosis) were not enrolled in this study.

### Generation of Peptide Pools

Peptides for all the antigens tested in the study were synthesized by A&A, LLC (San Diego) on a small scale (1 mg/ml). The APP peptide pool (total of 153 peptides, 15-mers overlapping by 10 residues spanning the entire protein sequence), Aβ (9 15-mer peptides spanning the Aβ-42 peptide), α-syn epitope pool (total of 11 peptides) (Sulzer et al., 2017), tau (total of 70 peptides) (Lindestam Arlehamn et al., 2019), TDP-43 peptide pool (total of 82 peptides, 15-mers overlapping by 10 residues spanning the entire protein sequence), pertussis (PT) (total of 132 peptides) (Bancroft et al., 2016; da Silva Antunes et al., 2018), and EBV/CMV pool (total of 270 peptides) (Dan et al., 2016; Tian et al., 2017) were synthesized and then reconstituted in DMSO. The individual peptides were then pooled, lyophilized and reconstituted at a concentration of 1 mg/ml. The peptide pools were tested at a final concentration of 2 or 5 ug/ml.

### Isolation of PBMCs

Whole blood was collected in EDTA vacutainers and PBMCs were isolated by density gradient centrifugation with Ficoll-Paque plus (GE #17144003). Briefly, blood was spun at 1850 rpm for 15 min with brakes off to remove plasma. Blood was then diluted with RPMI and 35 ml of blood was gently layered on 15 ml Ficoll-Paque plus and centrifuged at 1850 rpm for 25 min with brakes off. The cells at the interface were collected, washed with RPMI, counted and cryopreserved in 90% v/v FBS and 10 % v/v DMSO and stored in liquid nitrogen.

### Fluorospot Assay

Peripheral blood mononuclear cells were thawed and stimulated for 2 weeks *in vitro* at 2x10^6^ cells per well in a 24-well plate with APP, amyloid beta (Aβ), α-syn, tau, TDP-43, pertussis (PT), or EBV/CMV pools. PHA was used as control. Cells were fed with 10 U/ml recombinant IL-2 at an interval of 4 days. After 2 weeks of culture, cells were harvested and T cell responses to specific antigens were measured by IFNγ, IL-5, and IL-10 Fluorospot assay. Plates (Mabtech, Nacka Strand, Sweden) were coated overnight at 4°C with an antibody mixture of mouse anti-human IFNγ clone (clone 1-D1K), mouse anti-human IL-5 (clone TRFK5), and mouse anti-human IL-10 (clone 9D7). Briefly, 100,000 cells were plated in each well of the pre-coated Immobilon-FL PVDF 96 well plates (Mabtech), stimulated with the respective antigen at the respective concentration of 5 μg/ml and incubated at 37°C in a humidified CO_2_ incubator for 20−24 h. Cells stimulated with each antigen was also stimulated with 10 μg/ml PHA that served as a positive control. In order to assess nonspecific cytokine production, cells were also stimulated with DMSO at the corresponding concentration present in the peptide pools. All conditions were tested in triplicates. After incubation, cells were removed, plates were washed six times with 200 μl PBS/0.05% Tween 20 using an automated plate washer. After washing, 100 μl of an antibody mixture containing IFNγ (7-B6-1-FS-BAM), IL-5 (5A10-WASP), and IL-10 (12G8-biotin) prepared in PBS with 0.1% BSA was added to each well and plates were incubated for 2 h at room temperature. The plates were again washed six times as described above and incubated with diluted fluorophores (anti-BAM-490, anti-WASP-640, and SA-550) for 1 h at room temperature. After incubation, the plates were again washed as described above and incubated with a fluorescence enhancer for 15 min. Finally, the plates were blotted dry and spots were counted by computer-assisted image analysis (AID iSpot, AID Diagnostica GMBH, Strassberg, Germany). The responses were considered positive if they met all three criteria (i) the net spot forming cells per 10^6^ PBMC were ≥100 (ii) the stimulation index ≥2, and (iii) *p* ≤ 0.05 by Student’s *t*-test or Poisson distribution test.

### Proliferation Assay

Peripheral blood mononuclear cells were thawed in RPMI supplemented with 5% human serum (Gemini Bio-Products, West Sacramento, CA), 1% Glutamax (Gibco, Waltham, MA, United States), 1% penicillin/streptomycin (Omega Scientific, Tarzana, CA, United States), and 50 U/ml Benzonase (Millipore Sigma, Burlington, MA, United States). The cells were then washed and viable cells were counted using the trypan blue dye exclusion method. Viable cells were labeled with 5-chloromethylfluorescein diacetate (CFSE) at a concentration of 10 uM by incubating the cells suspended in 1 ml of PBS with CFSE at 37°C for 10−12 min. The labeled cells were then washed twice with 20% FBS prepared in PBS and spun at 2500 rpm for 5 min. Cells were then resuspended and cultured for 11 days in RPMI media supplemented with 5% human Ab serum, Glutamax and penicillin/streptomycin in the presence of APP, amyloid beta, α-syn, tau, TDP-43, EBV/CMV and PT peptide pools. After 4 days of culture cells were supplemented with 10 U/ml recombinant IL-2 and on day 8, cells were again stimulated with peptide pools. On day 11, cells were stained with a mix of anti- CD3-AF700 (clone UCHT1, BD pharmigen), anti-CD4-APC ef 780 (clone RPA-T4, eBiosciences) and anti-CD8a-BV650 (clone RPA-T8, Biolegend). The percentage of proliferating CD3+ CD4+ T cells was used a read out. The samples were acquired on BD LSR I flow cytometer (BD Biosciences, San Jose, CA). The percentage of proliferating CD3+ CD4+CFSE-T cells to each antigen was calculated by subtracting the background values (as determined from DMSO stimulated control) using FlowJo X Software (FlowJo LLC, Ashland, OR, United States). The gating strategy is shown in Supplementary Figure S3.

### Flow Cytometry

Cryopreserved PBMCs were thawed in RPMI supplemented with 5% human serum (Gemini Bio-Products, West Sacramento, CA, United States), 1% Glutamax (Gibco, Waltham, MA, United States), 1% penicillin/streptomycin (Omega Scientific, Tarzana, CA, United States), and 50 U/ml Benzonase (Millipore Sigma, Burlington, MA, United States). Cells were then washed and counted. 1 million cells were then blocked in 10% FBS for 10 min at 4°C. After blocking, cells were stained with a combination of APCef780 conjugated anti-CD4 (clone RPA-T4, eBiosciences), AF700 conjugated anti-CD3 (clone UCHT1, BD Pharmigen), BV650 conjugated anti-CD8a (clone RPA-T8, Biolegend), PECy7 conjugated anti-CD19 (clone HIB19, TONBO), APC conjugated anti-CD14 (clone 61D3, TONBO), PerCPCy5.5 conjugated anti-CCR7 (clone G043H7, Biolegend), PE conjugated anti-CD56 (eBiosciences), FITC conjugated anti-CD25 (clone M-A251, BD Pharmigen), eF450 conjugated anti-CD45RA (clone HI100, eBiosciences) and eF506 live dead aqua dye (eBiosciences) for 30 min at 4°C. Cells were then washed twice and acquired or sorted on a BD FACSAria flow cytometer (BD Biosciences, San Jose, CA) to measure the frequency of different cell subsets. The gating strategy is shown in Supplementary Figure S1.

### RNA Extraction and cDNA Library Preparation

A total of 100,000 PBMCs, CD4 or CD8 memory T cells were sorted and collected in Trizol in a 1.5 ml tube. Memory T cells were sorted based on CD45RA and CCR7 expression (Supplementary Figure S1), where CD45RA^–^CCR7^+^ Tcm, CD45RA^–^CCR7^–^ Tem, and CD45RA^+^CCR7^–^ Temra cells were included and CD45RA^+^CCR7^+^ naïve T cells were excluded. Tubes were vortexed, spun and stored at −80°C until processed. RNA was extracted using miRNeasy Micro Kit (Qiagen) on a Qiacube. Purified total RNA was amplified following the smart-seq2 protocol. cDNA was purified using AMPure XP beads, and barcoded Illumina sequencing libraries were generated, loaded and sequenced on the Illumina sequencing platform HiSeq 2500.

### RNAseq Analysis

Samples were sequenced using Hiseq 2500 (Illumina) to obtain 50 bp single reads. The paired-end reads that passed Illumina filters were filtered for reads aligning to tRNA, rRNA, adapter sequences, and spike-in controls. The reads were then aligned to GRCh38 reference genome and Gencode v27 annotations, which includes protein coding genes as well as pseudogenes, lncRNAs, and miRNAs, using STAR (v2.6.1c) (Dobin et al., 2013). DUST scores were calculated with PRINSEQ Lite (v 0.20.3) (Schmieder and Edwards, 2011) and low-complexity reads (DUST > 4) were removed from the BAM files. The alignment results were parsed via the SAMtools (Li et al., 2009) to generate SAM files. Read counts of each genomic feature were obtained with the featureCounts program (v1.6.5) (Liao et al., 2014). After removing absent features (zero counts in all samples), the raw counts were then imported to R/Bioconductor package DESeq2 (Love et al., 2014) to identify differentially expressed genes among samples. *P*-values for differential expression were calculated using the Wald test for differences between the base means of two conditions. These *p*-Values are then corrected for multiple tests using the Benjamini Hochberg algorithm (Benjamini and Hochberg, 1995) to control the false discovery rate. We considered genes differentially expressed between two groups of samples when the DESeq2 analysis resulted in an adjusted *P*-value of <0.05 and the absolute value of the log fold-change in gene expression was greater than 0.5. Variance stabilizing transformation (DESeq2 1.26.0) was applied on the read counts for all samples. Since there were samples from multiple mapping runs, an adjustment for batch effects using the outcome of interest as the disease state was performed using ComBat (sva 3.34.0). A principal components analysis was performed on the top 1000 variable genes using the prcomp function in the stats v3.6.3 library under R v3.6.3. The RNA-seq dataset analyzed as part of this study have been deposited in the NCBI Gene Expression Omnibus (GEO) database with the primary accession number GSE153104.

### Statistics

Statistical analyses were performed using GraphPad Prism version 8.1.1. Data were analyzed using non-parametric statistical tests. Mann−Whitney two-tailed test was performed to compare T cell responses to all the antigens and frequency of cell subsets in AD and HC. Spearman test was performed to check the significance of correlation between T cell responses and clinical variables.

## Data Availability Statement

The RNA-seq dataset analyzed as part of this study have been deposited in the NCBI Gene Expression Omnibus (GEO) database with the primary accession number GSE153104.

## Ethics Statement

The studies involving human participants were reviewed and approved by the La Jolla Institute for Immunology (LJI; protocol number VD-155) and the Columbia University Medical Center (CUMC; protocol number IRB-AAAQ9714). The patients/participants provided their written informed consent to participate in this study.

## Author Contributions

RD, BP, DS, AS, and CL designed and directed the study. RD and JP performed the experiments and analyzed the data. RD and AP analyzed the RNA-seq data. FB, MP, and AK provided the crucial reagents. JD and KM recruited the participants and performed the clinical evaluations. AF maintained the patient data, records, and assisted in participant recruitment. RD, DS, AS, and CL wrote the manuscript. All authors read, edited, and approved the manuscript.

## Conflict of Interest

The authors declare that the research was conducted in the absence of any commercial or financial relationships that could be construed as a potential conflict of interest.
