# Corrigendum: T Cell Responses to Neural Autoantigens Are Similar in Alzheimer's Disease Patients and Age-Matched Healthy Controls

**DOI:** 10.3389/fnins.2020.641809

**Published:** 2021-01-13

**Authors:** Rekha Dhanwani, John Pham, Ashmitaa Logandha Ramamoorthy Premlal, April Frazier, Atul Kumar, Maria Elena Pero, Francesca Bartolini, Juliana Rezende Dutra, Karen S. Marder, Bjoern Peters, David Sulzer, Alessandro Sette, Cecilia S. Lindestam Arlehamn

**Affiliations:** ^1^Division of Vaccine Discovery, La Jolla Institute for Immunology, La Jolla, CA, United States; ^2^Department of Pathology and Cell Biology, Columbia University, New York, NY, United States; ^3^Department of Veterinary Medicine and Animal Production, University of Naples Federico II, Naples, Italy; ^4^Department of Neurology, Columbia University Irving Medical Center, New York, NY, United States; ^5^Department of Medicine, University of California, San Diego, La Jolla, CA, United States; ^6^Department of Neurology, New York State Psychiatric Institute, Columbia University, New York, NY, United States; ^7^Department of Psychiatry and Pharmacology, New York State Psychiatric Institute, Columbia University, New York, NY, United States

**Keywords:** Alzheimer's disease, neurodegenration, autoimmunity, T cell responses, transcriptomics, neuroantigens

In the original article, we neglected to include the funder NIH/NIA, P30 AG066462 to KM.

The authors apologize for this error and state that this does not change the scientific conclusions of the article in any way. The original article has been updated.

