# Supplementary material for: T Cell Responses to Neural Autoantigens Are Similar in Alzheimer’s Disease Patients and Age-Matched Healthy Controls
Source: Front Neurosci. 2020 Aug 27;14:874. doi: 10.3389/fnins.2020.00874 (PMC7481378; doi:10.3389/fnins.2020.00874)
Supplement: Supplementary file 1 [file Data_Sheet_1.pdf]

Supplemental material

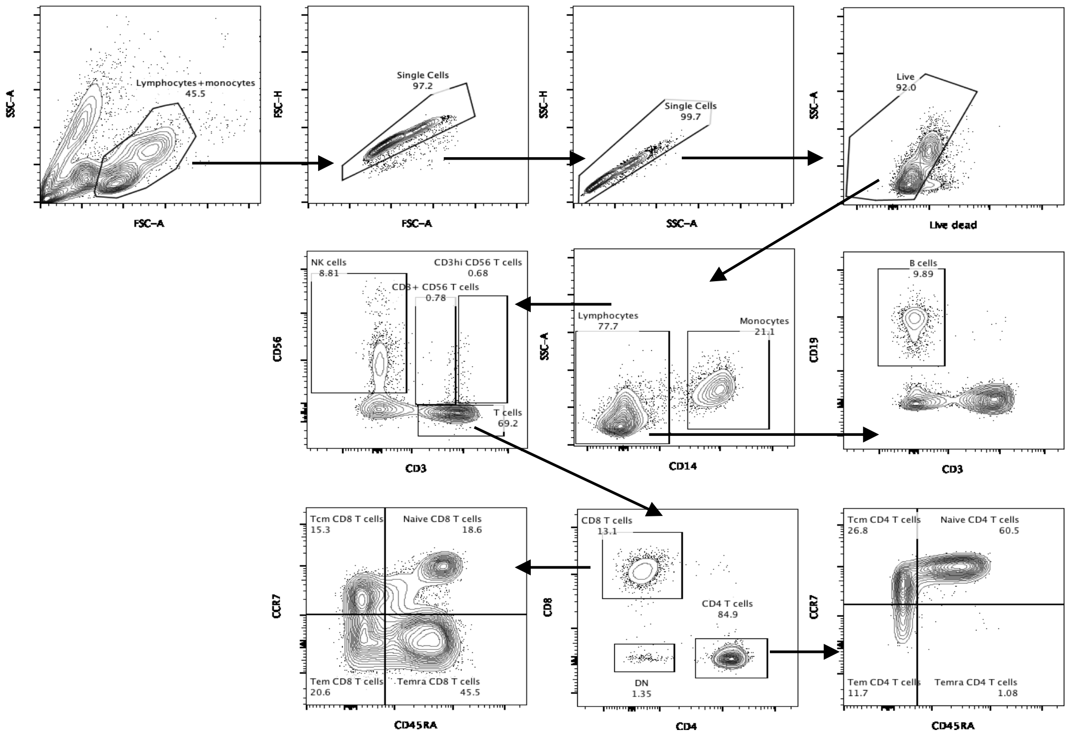

Fig S1. Gating strategy for each cell subset population.

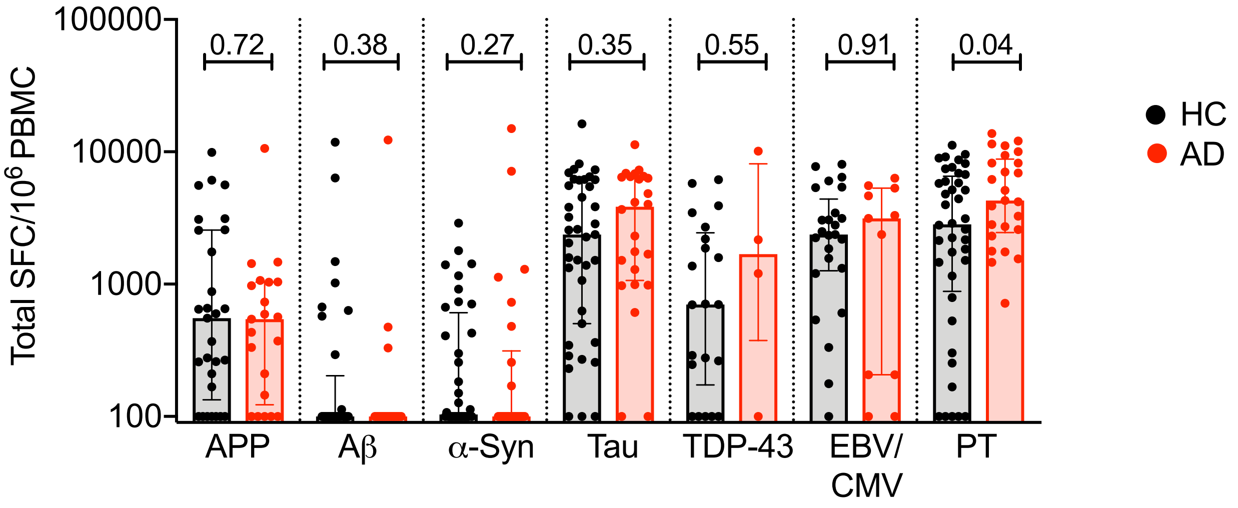

Fig S2. T cell reactivity to APP, Aβ, α-synuclein, tau, TDP-43, EBV/CMV, and PT in Caucasian AD and age-matched HC subjects. Magnitude of total response (sum of IFNγ, IL-5 and IL-10) in HC (black bar and circles) and AD (red bar and circles) to APP (HC, n=29; AD n=21), Aβ (HC, n=33; AD, n=19), α-syn (HC, n=36; AD, n=26), tau (HC, n=39; AD n=24), TDP-43 (HC, n=21; AD, n=4), EBV/CMV (HC, n=25; AD, n=11), and PT (HC, n=40; AD, n=25). Each dot represents a subject. Median ± interquartile range is shown. Two-tailed Mann-Whitney test.

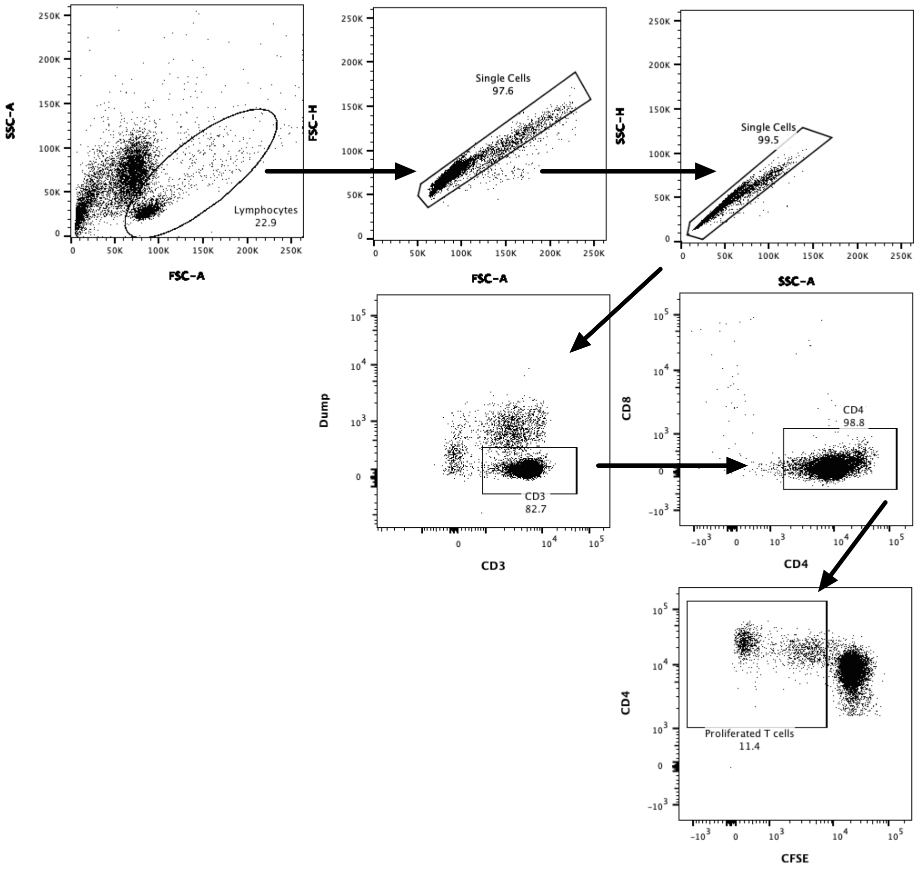

Fig S3. **Gating strategy adopted to measure the percentage proliferated CD4$^+$ T cells.** CD4$^+$CFSE$^-$ T cells represented the proliferated CD4$^+$ T cells upon stimulation.

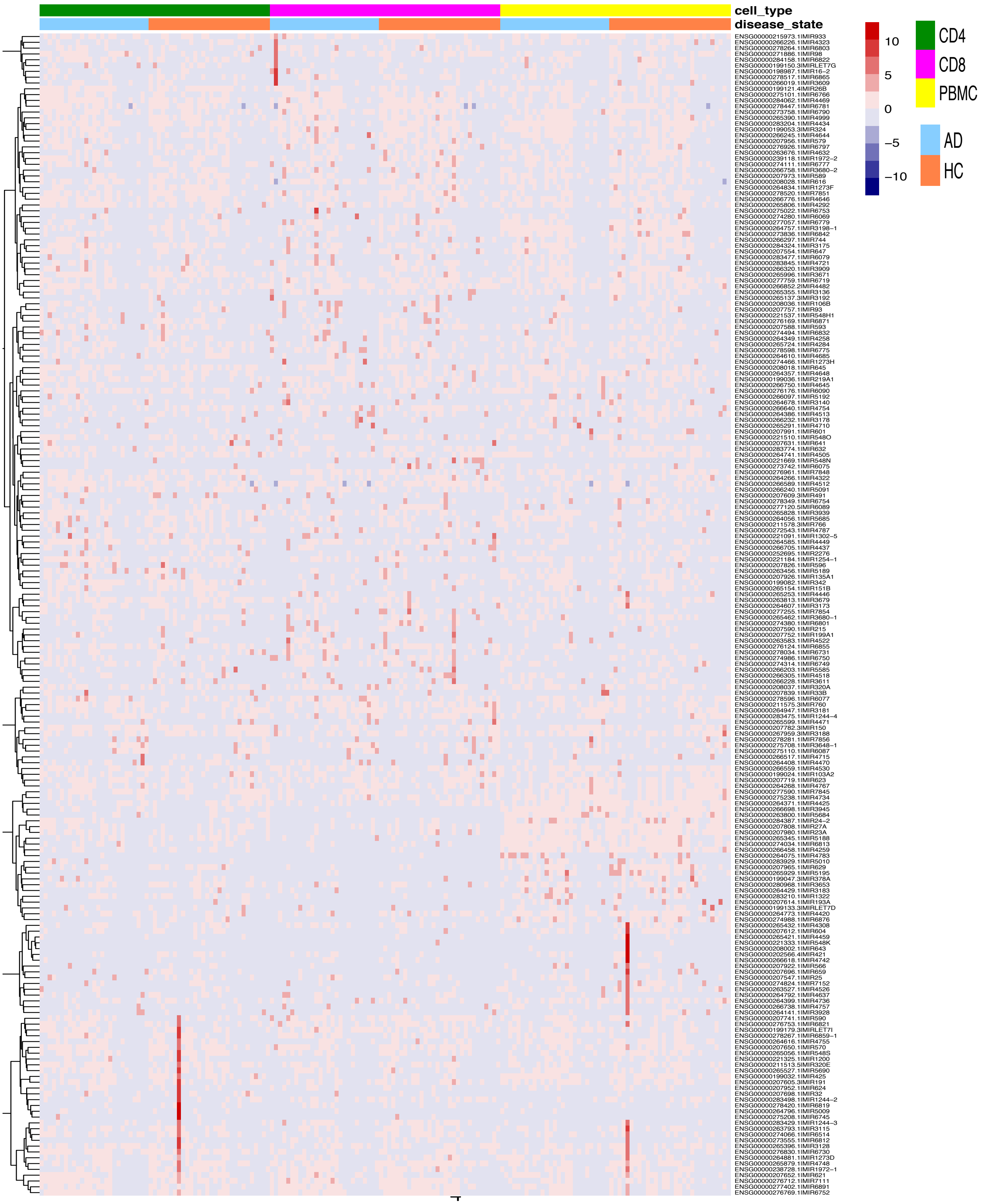

cell_type
disease_state
10
5
0
−5
−10
CD4
CD8
PBMC
AD
HC

Fig S4. Heat map of top 200 variable miRNAs in PBMCs (yellow bar), CD4 memory (green bar) and CD8 memory (pink bar) T cell subsets showing no difference in expression of miRNAs between AD (blue bar) and HC subjects (orange bar).

Table S1. Top 100 variable genes across PBMCs, CD4 and CD8 memory T cells.

No.	Gene ID	Gene name	CD4 memory T cells (AD vs. HC)		CD8 memory T cells (AD vs. HC)		PBMC (AD vs. HC)	
			Log2FC	Padj	Log2FC	Padj	Log2FC	Padj
1	ENSG00000224397.5	SMIM25	-0.42	0.77	0.44	0.95	-0.02	1
2	ENSG00000110077.14	MS4A6A	-0.33	0.84	0.01	1	0.11	1
3	ENSG00000112799.8	LY86	-0.36	0.81	0.32	1	-0.04	1
4	ENSG00000197249.13	SERPINA1	-0.25	0.89	0.09	1	-0.07	1
5	ENSG00000158869.10	FCER1G	-0.25	0.9	0.12	1	0.16	1
6	ENSG00000204472.12	AIF1	-0.26	0.88	0.05	1	-0.08	1
7	ENSG00000163220.10	S100A9	-0.2	0.93	0.08	1	0	1
8	ENSG00000143546.9	S100A8	-0.32	0.86	0.01	1	0.04	1
9	ENSG00000163221.8	S100A12	-0.28	0.87	-0.01	1	0.02	1
10	ENSG00000101439.8	CST3	-0.38	0.8	-0.01	1	-0.08	1
11	ENSG00000124731.12	TREM1	-0.02	0.99	-0.29	1	0.19	1
12	ENSG00000170458.13	CD14	-0.07	0.98	-0.21	1	0.08	1
13	ENSG00000090382.6	LYZ	-0.42	0.77	0.07	1	0.3	1
14	ENSG00000085265.10	FCN1	-0.19	0.91	0.2	1	0.13	1
15	ENSG00000119535.17	CSF3R	-0.23	0.9	-0.2	1	-0.03	1
16	ENSG00000121316.10	PLBD1	-0.26	0.87	-0.08	1	0.07	1
17	ENSG00000175489.9	LRRC25	-0.23	0.9	-0.03	1	-0.13	1
18	ENSG00000066336.11	SPI1	-0.22	0.9	-0.11	1	0	1
19	ENSG00000172243.17	CLEC7A	-0.22	0.92	-0.13	1	0.13	1
20	ENSG00000133246.11	PRAM1	-0.38	0.79	-0.59	0.047	0.11	1
21	ENSG00000186818.12	LILRB4	-0.27	0.83	-0.64	1	0.07	1
22	ENSG00000165168.7	CYBB	-0.21	0.91	-0.05	0.67	0.01	1
23	ENSG00000197629.5	MPEG1	-0.2	0.92	-0.03	1	-0.01	1
24	ENSG00000143226.13	FCGR2A	-0.12	0.95	0.07	1	0.06	1
25	ENSG00000085514.15	PILRA	-0.22	0.91	0.18	1	-0.02	1
26	ENSG00000187116.13	LILRA5	-0.12	0.96	0.36	1	0.05	1
27	ENSG00000111275.12	ALDH2	-0.3	0.86	0.08	1	-0.17	1
28	ENSG00000106066.14	CPVL	-0.27	0.88	-0.03	1	-0.09	1
29	ENSG00000131042.14	LILRB2	-0.17	0.93	0.37	1	0.15	1
30	ENSG00000111321.10	LTBR	-0.13	0.94	0.34	1	-0.04	1
31	ENSG00000158825.5	CDA	-0.26	0.87	0.1	1	0.09	1
32	ENSG00000214212.8	C19orf38	-0.15	0.94	0.33	1	0.01	1
33	ENSG00000038427.15	VCAN	-0.25	0.9	-0.12	1	0.1	1
34	ENSG00000239998.5	LILRA2	-0.18	0.92	0.18	1	0.06	1
35	ENSG00000161955.16	TNFSF13	-0.24	0.87	0.13	1	0	1
36	ENSG00000101336.13	HCK	-0.24	0.89	0.01	1	0.03	1
37	ENSG00000161642.17	ZNF385A	-0.31	0.85	0.1	1	-0.01	1
38	ENSG00000235568.6	NFAM1	-0.22	0.9	0.03	1	-0.15	1
39	ENSG00000186074.18	CD300LF	-0.07	0.98	-0.04	1	-0.1	1
40	ENSG00000198053.11	SIRPA	-0.16	0.94	-0.03	0.96	-0.17	1
41	ENSG00000006534.15	ALDH3B1	-0.14	0.95	-0.17	1	-0.11	1
42	ENSG00000204103.3	MAFB	-0.41	0.79	0.19	1	-0.02	1
43	ENSG00000126759.13	CFP	-0.41	0.77	0.14	1	0.04	1
44	ENSG00000168461.12	RAB31	-0.42	0.79	0.1	1	0.08	1
45	ENSG00000135218.17	CD36	-0.28	0.86	0.15	1	0.19	1

46	ENSG00000169403.11	PTAFR	-0.29	0.87	0.05	1	-0.06	1
47	ENSG00000136826.14	KLF4	-0.22	0.91	0.05	1	0.18	1
48	ENSG00000123689.5	G0S2	-0.24	0.9	-0.08	1	-0.18	1
49	ENSG00000216490.3	IFI30	-0.55	0.66	0.06	1	-0.15	1
50	ENSG00000185215.8	TNFAIP2	-0.46	0.75	-0.27	1	-0.3	1
51	ENSG00000131401.11	NAPSB	-0.31	0.86	-0.06	1	0.04	1
52	ENSG00000197405.7	C5AR1	-0.02	1	-0.08	1	0.16	1
53	ENSG00000171051.8	FPR1	0.01	1	-0.64	0.39	0.14	1
54	ENSG00000155465.18	SLC7A7	-0.08	0.98	0.07	1	-0.01	1
55	ENSG00000165025.14	SYK	-0.13	0.96	0.16	1	0.05	1
56	ENSG00000100292.16	HMOX1	-0.12	0.96	0.43	0.76	-0.2	1
57	ENSG00000196576.14	PLXNB2	-0.06	0.98	-0.52	0.073	-0.24	1
58	ENSG00000120708.16	TGFBI	-0.15	0.95	0.13	1	-0.06	1
59	ENSG00000116701.14	NCF2	-0.17	0.94	-0.13	1	-0.05	1
60	ENSG00000161944.16	ASGR2	-0.34	0.81	-0.63	1	-0.06	1
61	ENSG00000135929.8	CYP27A1	-0.23	0.88	-0.64	0.24	-0.23	1
62	ENSG00000182511.11	FES	-0.3	0.82	0.31	1	0.2	1
63	ENSG00000160883.10	HK3	-0.13	0.97	-0.27	1	-0.03	1
64	ENSG00000146094.13	DOK3	-0.05	0.99	-0.02	1	-0.14	1
65	ENSG00000166428.12	PLD4	-0.24	0.9	-0.63	0.4	0.07	1
66	ENSG00000186407.6	CD300E	-0.14	0.95	0.65	0.25	-0.13	1
67	ENSG00000011422.11	PLAUR	-0.3	0.87	-0.15	1	-0.04	1
68	ENSG00000123384.13	LRP1	-0.06	0.98	0.41	1	0.1	1
69	ENSG00000171236.9	LRG1	-0.39	0.76	-0.01	1	-0.27	1
70	ENSG00000128383.12	APOBEC3A	-0.09	0.95	0.27	1	0.19	1
71	ENSG00000197766.7	CFD	-0.22	0.91	0.01	1	-0.12	1
72	ENSG00000100079.6	LGALS2	-0.2	0.92	0.22	1	0.11	1
73	ENSG00000152804.10	HHEX	-0.29	0.86	0.16	1	0	1
74	ENSG00000166825.13	ANPEP	-0.22	0.91	0	0.94	-0.05	1
75	ENSG00000175591.11	P2RY2	-0.22	0.89	0.16	1	-0.3	1
76	ENSG00000146192.14	FGD2	-0.68	0.59	-0.41	1	-0.21	1
77	ENSG00000128604.19	IRF5	-0.28	0.87	-0.07	1	-0.27	1
78	ENSG00000125538.11	IL1B	-0.42	0.79	0.36	1	-0.07	1
79	ENSG00000113749.7	HRH2	-0.25	0.89	-0.04	1	-0.16	1
80	ENSG00000140678.16	ITGAX	-0.24	0.91	0.37	1	0	1
81	ENSG00000254415.3	SIGLEC14	-0.17	0.92	0.29	0.52	0.08	1
82	ENSG00000173372.16	C1QA	-0.04	0.98	0.26	0.39	0.08	1
83	ENSG00000011600.11	TYROBP	-0.15	0.95	0	1	0.02	1
84	ENSG00000106565.17	TMEM176B	-0.5	0.68	0.37	1	-0.15	1
85	ENSG00000002933.7	TMEM176A	-0.43	0.72	0.03	1	-0.16	1
86	ENSG00000148344.10	PTGES	0	1	-0.29	1	-0.06	1
87	ENSG00000211899.9	IGHM	-0.03	0.99	0.38	1	1.04	1
88	ENSG00000211677.2	IGLC2	0.02	0.99	-0.06	1	1.27	1
89	ENSG00000211895.5	IGHA1	-0.59	0.86	-0.68	1	-0.21	1
90	ENSG00000211592.8	IGKC	-0.41	0.77	-0.19	1	-0.3	1
91	ENSG00000211898.7	IGHD	-0.34	0.8	0.21	1	0.2	1
92	ENSG00000105369.9	CD79A	0.06	0.98	-0.25	1	0.25	1
93	ENSG00000010610.9	CD4	0.03	0.99	-0.04	1	-0.13	1
94	ENSG00000244734.3	HBB	0.44	0.72	0.23	1	0.1	1
95	ENSG00000188536.12	HBA2	0.35	0.79	0.21	1	-0.03	1

96	ENSG00000129824.15	RPS4Y1	0.19	0.92	0.07	1	0.02	1
97	ENSG00000099725.14	PRKY	0.11	0.96	0.05	1	0.03	1
98	ENSG00000172116.21	CD8B	0.06	0.99	0	1	-0.34	1
99	ENSG00000153563.15	CD8A	0.48	0.76	-0.03	1	-0.39	1
100	ENSG00000100453.12	GZMB	0.42	0.79	0.15	1	0.39	1
